# Genetic Structure and Selection Signals in Hetian Gray Donkeys and Xinjiang Donkeys from Xinjiang, China

**DOI:** 10.3390/ani16182825

**Published:** 2026-09-08

**Authors:** Yongqing Li, Shihao Liu, Juan Geng, Weiwei Wu

**Affiliations:** 1 Xinjiang Academy of Animal Sciences, Urumqi 830011, China; m18894036031@163.com (Y.L.);; 2 Xinjiang Uygur Autonomous Region Sheep and Goat Engineering Research Center, Urumqi 830011, China; 3 Xinjiang Animal Husbandry General Station, Urumqi 830052, China

**Keywords:** Hetian Gray Donkeys, Xinjiang Donkeys, genetic structure, selection signals

## Abstract

The Hetian Gray Donkeys (China_HTQD) and Xinjiang Donkeys (China_XJD) are representative local donkey breeds in Xinjiang, China, that show significant differences in production phenotypes, such as body size and coat color. However, their genetic background and the molecular mechanisms influenced by artificial selection have not been systematically elucidated. This study integrated whole-genome data from 60 newly sequenced China_HTQD and China_XJD individuals, along with 155 global reference datasets from the NCBI database, to construct a modern donkey whole-genome variation set covering domestic and overseas populations (including Africa, West and Central Asia, and Europe). The results of genetic diversity analysis indicated that China_XJD has slightly higher genetic diversity than China_HTQD, while the China reference population (China_Ref) and European population (Europe) have the lowest genetic diversity. China_HTQD and China_XJD have greater genetic variation. Both PCA and NJ phylogenetic analyses showed that China_HTQD and China_XJD are genetically very close, clustering within the Chinese donkey population background, and exhibit clear geographic genetic isolation from overseas reference populations. ADMIXTURE analysis (with K = 2 showing the lowest cross-validation error) revealed that the two breeds share a highly similar genetic structure, with only minor differences in the proportions of specific ancestral components, while both are distinctly different from the other four reference populations. A whole-genome differentiation scan (*F*_st_) showed limited overall genetic differentiation between China_HTQD and China_XJD populations, but significant differentiation peaks were observed in some genomic regions. Based on the joint screening of windowed *F*_st_ (Fixation Index) and *XP-CLR* (Cross-Population Composite Likelihood Ratio), this study identified two candidate genomic regions containing the *TBX3* and *ZFAT* genes. Among these, *TBX3* emerged as a strong candidate with a robust selection signal and a well-established role in body size regulation. In contrast, *ZFAT* showed a weaker signal, and its potential involvement in coat color remains speculative and requires further validation. In summary, this study not only clarified the close genetic relationship and the level of interpopulation differentiation between China_HTQD and China_XJD at the whole-genome level but also identified candidate genes potentially associated with phenotypic divergence, providing gene-level confirmation of *TBX3* and the novel identification of *ZFAT* as a candidate for coat-color variation, thereby providing a scientific foundation for elucidating the genetic mechanisms underlying phenotypic differences in Xinjiang’s local donkey genetic resources.

## Simple Summary

Xinjiang, China, is home to two local donkey breeds: Hetian Gray Donkeys (HTQD) and Xinjiang Donkeys (XJD). They differ noticeably in body size and coat color, but the genetic basis of these differences has remained unclear. In this study, we sequenced the whole genomes of 60 donkeys from these two breeds and combined the data with 155 publicly available donkey genomes from other regions, including Africa, West/Central Asia, and Europe. We found that XJD has slightly higher genetic diversity than HTQD, while European and reference Chinese donkeys show the lowest diversity. Both Xinjiang breeds are genetically very close to each other but clearly separate from overseas populations. Although their overall genetic difference is small, we detected strong selection signals in specific genomic regions. Two genes stood out: *TBX3* (strong candidate for body size) and *ZFAT* (weaker signal, potential role in coat color requires validation). Our findings reveal a close genetic relationship between HTQD and XJD and identify candidate genes underlying phenotypic differences, providing a genetic foundation for conserving Xinjiang’s donkey resources.

## 1. Introduction

The domestic donkey (*Equus asinus*) is one of the most important domesticated animals in human history, playing an irreplaceable role in long-term agricultural production, transportation, and frontier livelihood systems [1,2]. With the transformation of modern animal husbandry, the use value of donkeys is gradually shifting from traditional draft work to the in-depth development of biomedical raw materials, specialty meat and dairy products, and local high-quality genetic resources [3]. These findings provide molecular insights into the genetic mechanisms underlying phenotypic differences between the two breeds.

Early research on the domestication, origin, and spread of donkeys suggested a close phylogenetic link to the African wild ass and indicated a multi-source or multi-stage domestication pattern [4]. Subsequent ancient DNA evidence further revealed the complexity of the different wild ass types involved in the domestication process, providing direct clues to the phylogenetic composition of modern donkey populations [5]. In China, ancient DNA studies have shown that the maternal lineage of Chinese domestic donkeys and their historical spread exhibit unique characteristics [6], a finding corroborated by inferred patterns of diffusion and exchange based on modern population genetic data [7]. In recent years, large-scale genomic studies have further refined the overall picture of donkey domestication and spread across time and space, offering a clearer comparative framework for the study of regional population formation and differentiation [8].

With the availability of high-quality reference genomes and extensive resequencing resources, it has become feasible to analyze the genetic structure and selection signals of domestic donkeys at the whole-genome level [9]. High-quality reference genome assemblies have laid the foundation for variant detection, gene annotation, and the localization of selection signals [10]. Meanwhile, indicators such as runs of homozygosity (ROH) have been widely used to assess population genetic diversity and inbreeding history, providing quantitative evidence for understanding population events during breed formation [11]. In studies of Chinese domestic donkeys, several resequencing projects have examined the genetic structure, environmental adaptation, and selection signals across different local donkey populations [12,13,14]. Moreover, genomic studies on donkey domestication and body size-related traits have suggested that the breeding process has left detectable selection signatures in a few key genomic regions [15]. These studies collectively indicate that Chinese local donkey breeds have distinct regional genetic structures. However, detailed comparative analyses of closely related populations within finer geographic units (such as different local types within Xinjiang) are still relatively lacking [15,16].

Located in the heart of the Eurasian continent, Xinjiang has historically been a pivotal hub on the Silk Road, and its livestock populations often exhibit complex genetic inputs from multiple sources [17,18,19]. Meanwhile, the arid desert environment and specific production demands have imposed continuous selective pressures. Among local Xinjiang donkey breeds, Hetian Gray Donkeys (China_HTQD) and Xinjiang Donkeys (China_XJD) are highly representative. The Xinjiang donkey is an ancient indigenous breed tracing back to the 3rd century AD, originating from the Asian wild ass and developed through long-term domestication in southern Xinjiang [20]. The Hetian Gray donkey, in contrast, was officially recognized as a distinct breed in 2006, following approximately 200 years of selective breeding by local farmers for superior reproductive and milk performance, with recent genomic evidence suggesting contributions from both Xinjiang and Guanzhong donkeys to its formation [21]. While Li et al. [21] clarified the ancestral origins and genetic relationships of Chinese donkeys, the molecular mechanisms underlying phenotypic divergence—particularly body size and coat color—between Hetian Gray and Xinjiang donkeys remain largely unexplored. To address this gap, a systematic evaluation of the following aspects is urgently needed: the genetic positioning of these two breeds in a global reference context, the degree of fine-scale genetic differentiation between them, and candidate selection regions related to phenotypic differences still lack systematic assessment [22,23]. Since closely related populations typically have low overall differentiation, relying on a single statistic can easily yield unstable detection results. Therefore, combining a strictly quality-controlled dataset and conducting cross-validation through population structure, differentiation indices, and cross-population selection scans is a more robust research strategy [24,25].

Based on this, this study conducted whole-genome resequencing of 30 Xinjiang Donkeys (China_XJD) and 30 Hetian Gray Donkeys (China_HTQD), and integrated genomic data from globally representative populations in the NCBI public database to construct a comprehensive modern donkey variation dataset. Based on strict quality control, methods such as PCA, NJ phylogenetic tree, and ADMIXTURE were used to analyze population genetic structure and composition [26], and LD decay and diversity indices were combined to assess differences in genetic background. Furthermore, using China_HTQD and China_XJD as core comparison objects, *F*_st_ (Fixation Index) and *XP-CLR* (Cross-Population Composite Likelihood Ratio) were used for inter-population selection signal analysis [27,28], and the reproducibility of the process was ensured within a unified analytical framework [29]. This study aims to clarify the genetic positioning and differentiation patterns of local Xinjiang donkeys within a global reference framework, and to identify robust candidate genes associated with phenotypic differences between the two, providing molecular clues and a data basis for the conservation evaluation and subsequent precise breeding of local Xinjiang donkey genetic resources.

## 2. Materials and Methods

### 2.1. Experimental Animals

The study population included 215 whole-genome resequencing samples of domestic donkeys (*Equus asinus*) for population genetic structure analysis of two local Xinjiang donkey breeds (China_HTQD and China_XJD). The sources and sample numbers for each population are shown in Table 1. Blood samples were collected from 60 donkeys by professional veterinarians, anticoagulated with EDTA, and stored at −20 °C, with 30 China_HTQD individuals from a breeding farm in Pishan County and 30 China_XJD individuals from a breeding farm in Qira County. Breed assignment was confirmed through farm archival records, individual origin information, and on-site verification. All selected individuals were unrelated, clinically healthy, and normally developed, with consistent management conditions across farms. Body size and coat color phenotypes were also recorded for 30 China_HTQD and 30 China_XJD individuals (Table 2). The body-weight variation among these populations is interpreted as a breed-level characteristic. All measured animals were male, aged 3–4.5 years, and all measurements were performed by the same trained personnel to ensure consistency. Differences in body size traits (BW, BH, BL, CC, and CaC) between China_HTQD and China_XJD were assessed using one-way analysis of variance (ANOVA), with significance set at *p* < 0.05. For traits showing significant overall differences, Tukey’s HSD post hoc test was applied for pairwise comparisons. All statistical analyses were performed using R software (version 4.5.3), and results are presented as means ± standard deviations (SDs). Sex, age, and farm/herd origin were included as covariates in the model to control for potential confounding effects.

Additionally, 155 whole-genome data of donkeys (Table S1) from different regions around the world were downloaded from the NCBI public database https://www.ncbi.nlm.nih.gov/ (accessed on 3 March 2026), covering 82 donkeys from other local Chinese populations (CL, GLD, HB, XZCM, Yangyuan), 40 from African populations (EGY, ETH, KEN—YPO, SOM), 10 from European populations (CYK—Islas Canarias, English W pure Irish), and 23 from West and Central Asian populations (IRA—D, KAZ, KYR, MON, YEM, TUK). Small reference populations (*n* < 5) were retained in PCA and ADMIXTURE as part of the West Central Asia regional group for contextual visualization, with interpretations concerning them made cautiously. All primary conclusions were derived from populations with *n* ≥ 10.

### 2.2. Genomic DNA Extraction and Library Preparation

Genomic DNA was extracted from the 60 blood samples using the Magbead HMW DNA Kit (Kangwei Century, Taizhou, China). The concentration of DNA samples was measured using a Qubit fluorometer (Thermo Fisher Scientific, Waltham, MA, USA). The integrity and purity of DNA samples were assessed by electrophoresis on a 1% agarose gel. Only qualified samples were used for library preparation. Fragment DNA using dsDNA Fragmentase, perform end repair and dA-tailing, and verify fragment size (300–500 bp) by 2% agarose gel electrophoresis. Ligate sequencing adapters, purify with magnetic beads, and quantify by Qubit. Amplify ligation products by PCR, size-select with magnetic beads, then assess library concentration and fragment size using Qubit, 2% agarose gel electrophoresis, and Qsep400 bioanalyzer. Denature linear libraries to single strands for circularization, digest uncircularized linear molecules, and quantify the single-stranded circular libraries by Qubit. Prepare DNA nanoballs (DNBs) by rolling circle amplification, quantify by Qubit, and proceed to sequencing.

### 2.3. Whole-Genome Resequencing, Data Quality Control, and Alignment

The study involved whole-genome resequencing of 60 blood samples (30 China_HTQD and 30 China_XJD) on the MGI DNBSEQ-T7 platform, with an average sequencing depth of approximately 10×. Additionally, genomic data for 155 donkeys from global reference populations were obtained from the NCBI public database, bringing the total number of donkey whole-genome data to 215 (sample composition is shown in Table 1). The raw sequencing data for all samples were quality-controlled using fastp v0.23.2 to remove adapter sequences and low-quality reads. The clean reads were then aligned to the reference genome ASM1607732v2 (GenBank ID: GCA_016077325.2) (https://www.ncbi.nlm.nih.gov/datasets/genome/GCF_016077325.2/, accessed on 3 March 2026) using BWA-MEM v0.7.17. Single-sample variant detection was performed using GATK version 4.2.5.0 HaplotypeCaller to generate gVCF files, which were then jointly genotyped across samples using GATK (version 4.2.5.0) GenotypeGVCFs to obtain the raw VCF file. SNPs were hard-filtered according to the GATK Best Practices (https://gatk.broadinstitute.org/, accessed on 3 March 2026), with filtering criteria as follows: retaining biallelic SNPs and filtering out those with “QD < 2.0, MQ < 40.0, FS > 60.0, SOR > 3.0, MQRankSum < −12.5, or ReadPosRankSum < −8.0”. PLINK2 (v2.00a5.10LM (5 Jan 2024)) was further used to filter sites and individuals, with parameters “--geno 0.05 --mind 0.05 --maf 0.05”. Post-filtering VCF statistics and population-level missingness are summarized in Tables S2 and S3, respectively. For population structure analysis, LD pruning was applied to the SNP set to obtain a relatively independent marker set.

### 2.4. Genetic Diversity Analysis

Genetic diversity analyses were performed using the high-quality SNP set obtained after MAF filtering (MAF ≥ 0.05) described in Section 2.3. Plink was used to calculate the observed heterozygosity (*Ho*) and expected heterozygosity (*He*) for each SNP locus within populations, and the averages were used to represent these values. The polymorphism information content (*PIC*) was also calculated for each SNP locus in each population. VCFtools v0.1.16 was employed to compute nucleotide diversity (*π*) based on a sliding window approach (window size = 50,000 bp; step = 10,000 bp), using a 50 kb window and a 10 kb step size. To ensure comparability of results, *π* was calculated for all populations using the same filtered set of variants and the same windowing strategy to obtain the genome-wide distribution of *π*. Using the same strategy, Tajima’s *D* was calculated with VCFtools v0.1.16 to test whether populations deviate from neutral evolutionary models due to natural selection. ROH analysis was performed using PLINK v1.90b4.6 with the following parameters: minimum ROH length of 500 kb, minimum of 50 SNPs, maximum gap of 1000 kb, SNP density of 1 SNP per 50 kb, 50 SNPs per sliding window, one heterozygous SNP, and five missing genotypes allowed per window, and a window threshold of 0.05. The analysis was performed on 30 autosomes.

$$\mathrm{Inbreeding}\;\mathrm{coefficient}\;\mathrm{based}\;\mathrm{on}\;\mathrm{ROH}:\;F_{\mathrm{ROH}}=\sum L_{\mathrm{ROH}}/L_{\mathrm{AUTO}}$$

where *L_ROH_* is the total length of autosomal ROH segments in an individual, and LAUTO is the total length of autosomes in the reference genome. Finally, *F_ROH_* and the length distribution of ROH were summarized and compared at the population level.

### 2.5. Population Genetic Structure Analysis (PCA, ADMIXTURE)

PLINK2 v2.00a5.10LM (5 Jan 2024) was used to filter the vcf file containing biallelic SNP genotyping data for missingness and minor allele frequency (MAF), with the following parameters: --geno 0.05, --maf 0.05, --max-alleles 2. LD pruning was then performed using the sliding window method (--indep-pairwise 300 30 0.2) to obtain a relatively independent set of markers. Further filtering of individuals and sites for missing data was conducted on the pruned data (--mind 0.05, --geno 0.05), and the first 30 principal components were calculated using PLINK2 (--pca 30) for comparative analysis of population genetic structure. VCF2Dis v1.52 was used to calculate pairwise genetic distances between samples from the VCF file, generating a distance matrix. FastME v2.1.6.4 was then used to construct a neighbor-joining tree from the distance matrix. ADMIXTURE v1.3.0 was used to perform maximum likelihood estimation of individual ancestry to assess population genetic structure between different breeds, with K set from 2 to 15 and cross-validation enabled (--cv = 10). The K value with the minimum cross-validation error was used as the primary explanatory model to generate stacked bar charts evaluating the genetic structure of different populations. Population differentiation was analyzed using the population differentiation statistic (*F*_st_), with Weir–Cockerham *F*_st_ estimates (--weir-*F*_st_-pop) calculated by VCFtools v0.1.16 in sliding windows (window size = 50 kb, step = 10 kb) to obtain the genome-wide distribution of windowed *F*_st_. Outlier windows were defined using the empirical quantile method (the top 0.1% of genome-wide windowed *F*_st_ values were considered as candidate differentiated regions).

### 2.6. Selection Signal Analysis

In this study, VCFtools (v0.1.14) was used to calculate fixation index (*F*_st_) values using a sliding window approach (window size: 50 kb, step size: 10 kb). The parameters for VCFtools were set as follows: “--*F*_st_-window-size 100,000 --*F*_st_-window-step 50,000”. Regions with *F*_st_ values in the top 1% were identified as candidate selection regions. Concurrently, the *XP-CLR* method [28] was employed for a directional comparison between Hetian Gray Donkeys (HTQD) and Xinjiang Donkeys (XJD), with China_XJD as the reference population and China_HTQD as the target population. The parameters were set as “-w1 0.005 200 2000 -p0 0.95” (genetic distance window: 0.005 Morgan, maximum SNPs per window: 200, grid size: 2000 bp, correlation threshold: 0.95). Composite likelihood ratio (CLR) scores were calculated for each position, and genomic regions with CLR values in the top 0.01% were defined as significant selection segments. The intersection of candidate regions identified by both *XP-CLR* and *F*_st_ methods was used to identify candidate genes by comparing with reference genome gene annotation intervals using BEDtools v2.27.1. Annotated genes were subjected to GO enrichment analysis using DAVID Bioinformatics Resources (https://david.ncifcrf.gov/, accessed on 3 March 2026) and KEGG pathway enrichment analysis using KOBAS 2.0 (http://kobas.cbi.pku.edu.cn/, accessed on 3 March 2026), with a false discovery rate-adjusted *p*-value of 0.05 as the significance threshold.

## 3. Results

### 3.1. Sequencing Data and Alignment Analysis with the Reference Genome

Quality control was performed on the raw data obtained from whole-genome resequencing of Hetian Gray Donkeys (China_HTQD) and Xinjiang Donkeys (China_XJD), yielding average read counts of 170,631,025 and 180,114,408 clean reads, respectively. Among the NCBI reference populations, the European group had the lowest clean read count, with 98,392,037 reads. The GC content was above 41% for all populations (Table 3). Sequencing reads from each population were aligned to the reference genome (GCA_016077325.2), with alignment rates of 95–98% overall, and 97% for China_HTQD and 98% for China_XJD. The data quality and alignment rates met the requirements for subsequent analysis. Quality control results are shown in Figure S1 (locus missing rate and MAF) and Figure S2 (sample missing rate, MAF, and heterozygosity).

### 3.2. Genetic Diversity Analysis Results

**Figure 1:**
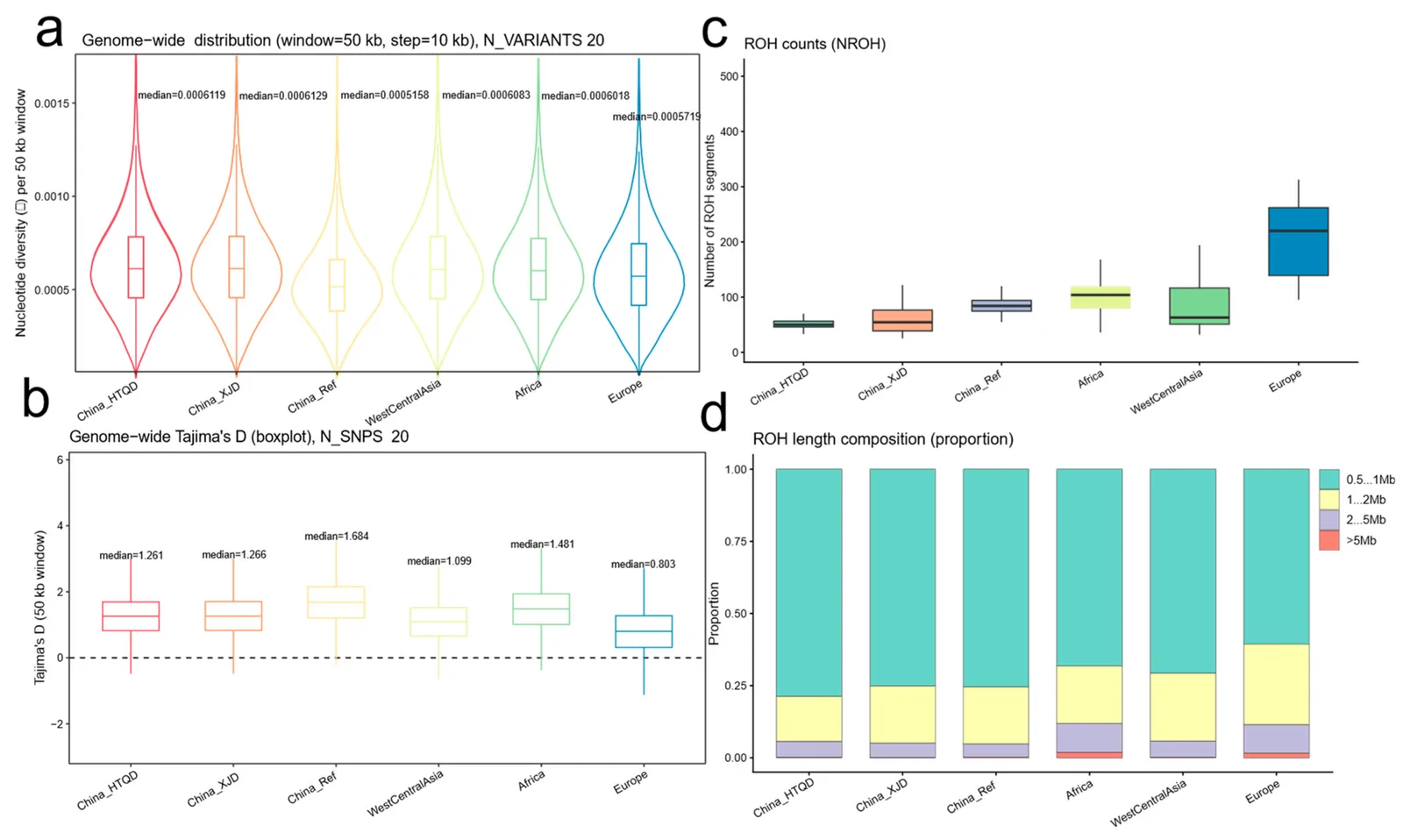
Analysis results of nucleotide diversity, neutrality test signals, and other genetic diversity indices, including runs of homozygosity (ROH), for each population: (**a**) nucleotide diversity (*π*); (**b**) Tajima’s *D*; (**c**) number of ROH segments (NROH); (**d**) length distribution of ROH.

The genetic parameters of the China_XJD population were slightly higher than those of the China_HTQD population. As shown in Table 4, the nucleotide diversity (*π*) values of the six populations (Figure 1a) were lowest in China_Ref and Europe, and highest in China_HTQD and China_XJD (which were almost identical). The median Tajima’s *D* values for the six populations ranged from 0.803 (Europe) to 1.68 (China_Ref), with values exceeding 1 observed in all populations except Europe. Notably, the European population showed the lowest median Tajima’s *D* among all groups (Figure 1b). These results suggest a potential deviation from neutrality, but should be interpreted with caution, as Tajima’s *D* alone does not provide formal statistical evidence without empirical significance testing, and values can be influenced by demographic history or population structure.

The two key ROH analysis indicators, median_NROH and median_TotalROH (Figure 1c,d), showed that the European population had much higher values than the other five populations. The West and Central Asian and African populations had intermediate levels. China_HTQD and China_XJD had relatively lower and similar NROH values, indicating low ROH accumulation. ROH fragments were predominantly short in all populations, but the proportion of medium- (and long)-length fragments varied: the European population had a higher proportion of longer ROH fragments, the West and Central Asian and African populations had intermediate proportions, and China_HTQD and China_XJD had mostly short ROH fragments with similar length distributions, consistent with their lower NROH results.

As shown in Table 4, the polymorphism information content (*PIC*) was similar between China_HTQD and China_XJD, while the European population had the highest *PIC* value (0.27189). The West and Central Asian population had the lowest observed heterozygosity (*Ho* = 0.077307), whereas the European population had the highest (*Ho* = 0.113682). The *Ho* values were lower than the expected heterozygosity (*He*) in all six populations, indicating inbreeding or population substructure. China_XJD had slightly higher *He* and *Ho* values than China_HTQD, suggesting that the genetic diversity of Xinjiang Donkeys (China_XJD) was higher than that of Hetian Gray Donkeys (China_HTQD).

### 3.3. Population Genetic Structure Analysis Results

**Figure 2:**
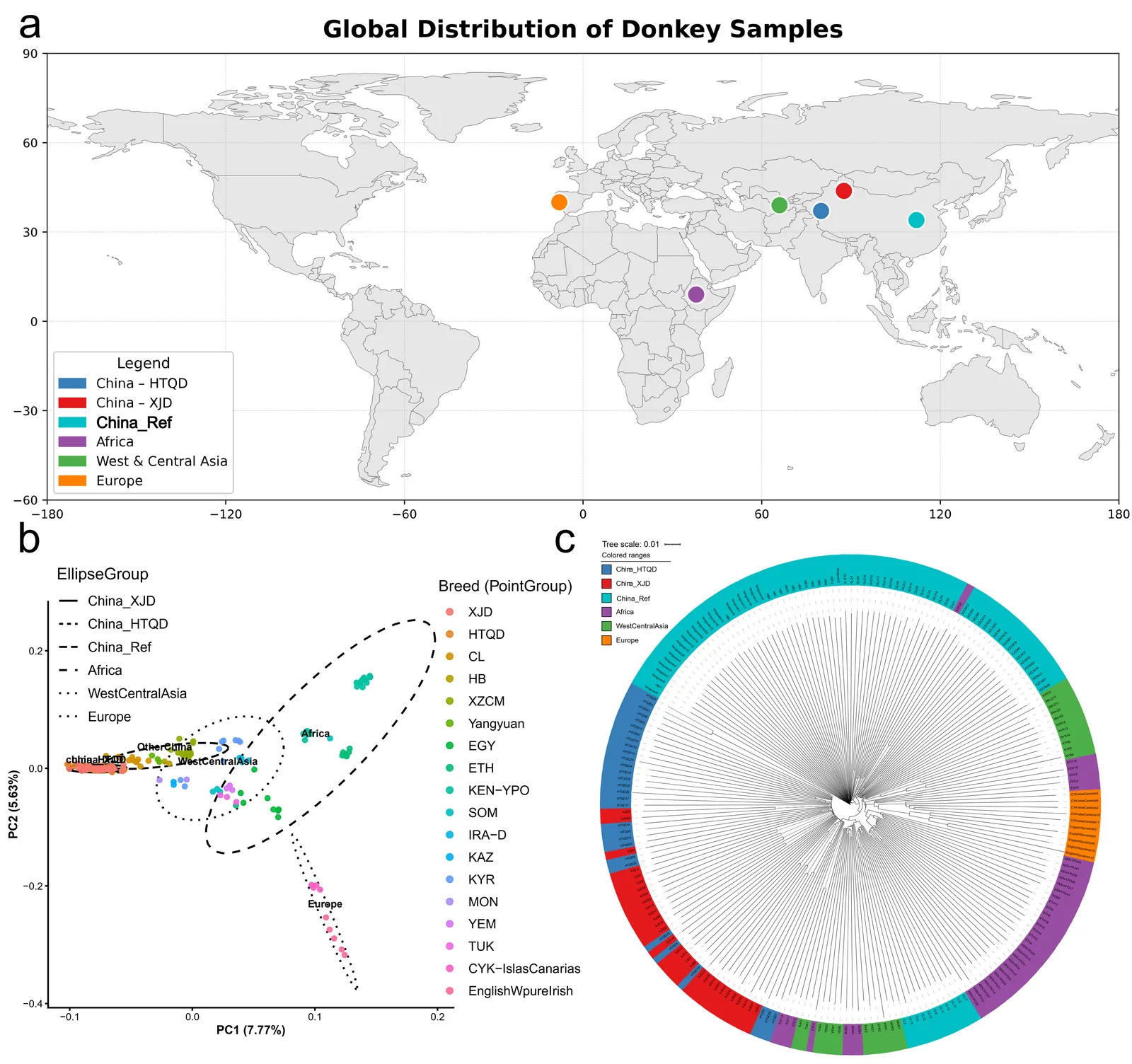
Geographic Sampling and Population Genetic Structure Analysis Results of the Donkey Dataset: (**a**) distribution of the origins of the study populations; (**b**) PCA plot; (**c**) phylogenetic tree.

After SNP quality control and filtering, the whole-genome data of 215 donkeys yielded a total of 96,892,950 SNPs. For PCA and NJ tree construction, LD pruning was performed to obtain a relatively independent set of markers, resulting in 87,991,070 SNPs for these analyses. PCA results are shown in Figure 2b. The analysis indicated that the six populations were roughly divided into four clusters, which corresponded to their geographical distributions. China_HTQD and China_XJD had the closest genetic distance, with most individuals overlapping in distribution. All Chinese populations were clustered within a single range. The African and European populations (Europe) showed relatively distant genetic relationships with the other five populations. The European population (Europe) was completely independently clustered, showing the greatest genetic distance and the most significant structural differences. The West and Central Asian populations exhibited a certain degree of genetic differentiation. The PCA results were consistent with the phylogenetic tree results (Figure 2c) and further corroborated the genetic differentiation pattern among populations, as analyzed using the inter-population genetic differentiation index.

**Figure 3:**
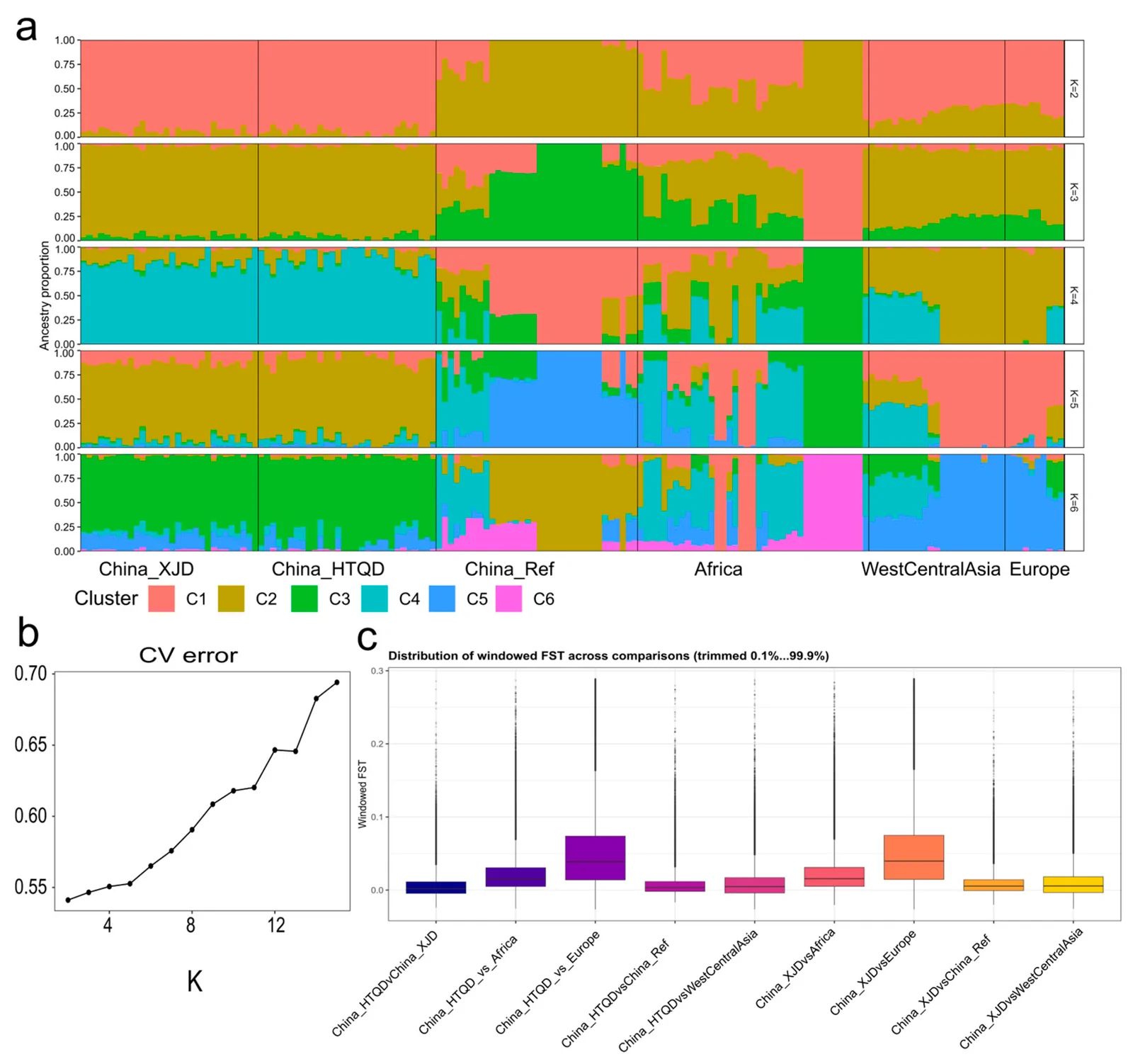
Results of ADMIXTURE and *F*_st_ Analysis: (**a**) ADMIXTURE (K = 2–6); (**b**) cross-validation curve of CV error; (**c**) distribution of Windowed *F*_st_.

Based on the ADMIXTURE v1.3.0 ancestry analysis results (Figure 3a,b) performed on the LD-pruned SNP set of 87,991,070 SNPs, K = 2 showed the lowest CV error and was therefore selected as the primary model. The results showed that the genetic composition of Xinjiang Donkeys (China_XJD) and Hetian Gray Donkeys (China_HTQD) was highly similar, with visible differences in the proportion of certain genetic components. These two populations also differed significantly from the European and African populations, suggesting shared ancestral components or potentially limited gene flow among them. The two Xinjiang populations (China_XJD and China_HTQD) had the greatest genetic differences from the other three populations (Africa, West Central Asia, and Europe), while some degree of shared ancestry was observed among all six populations. Pairwise genome-wide *F*_st_ values among all populations are provided in Table S4. The highest *F*_st_ values (0.051–0.052) were observed between China_XJD and Europe, and between China_HTQD and Europe, falling within the low range of genetic differentiation for domestic populations. The lowest *F*_st_ value (0.005) was found between the two Xinjiang populations (China_XJD and China_HTQD), consistent with the PCA and ADMIXTURE results, although local differentiation peaks were detected at specific genomic regions. The LD decay results (Figure S3) showed differences among the six populations’ curves, reflecting varying strengths of linkage disequilibrium among them. The curves of China_HTQD and China_XJD were close to each other and comparable to those of the other populations.

### 3.4. Inter-Population Selection Signal Analysis Results

**Figure 4:**
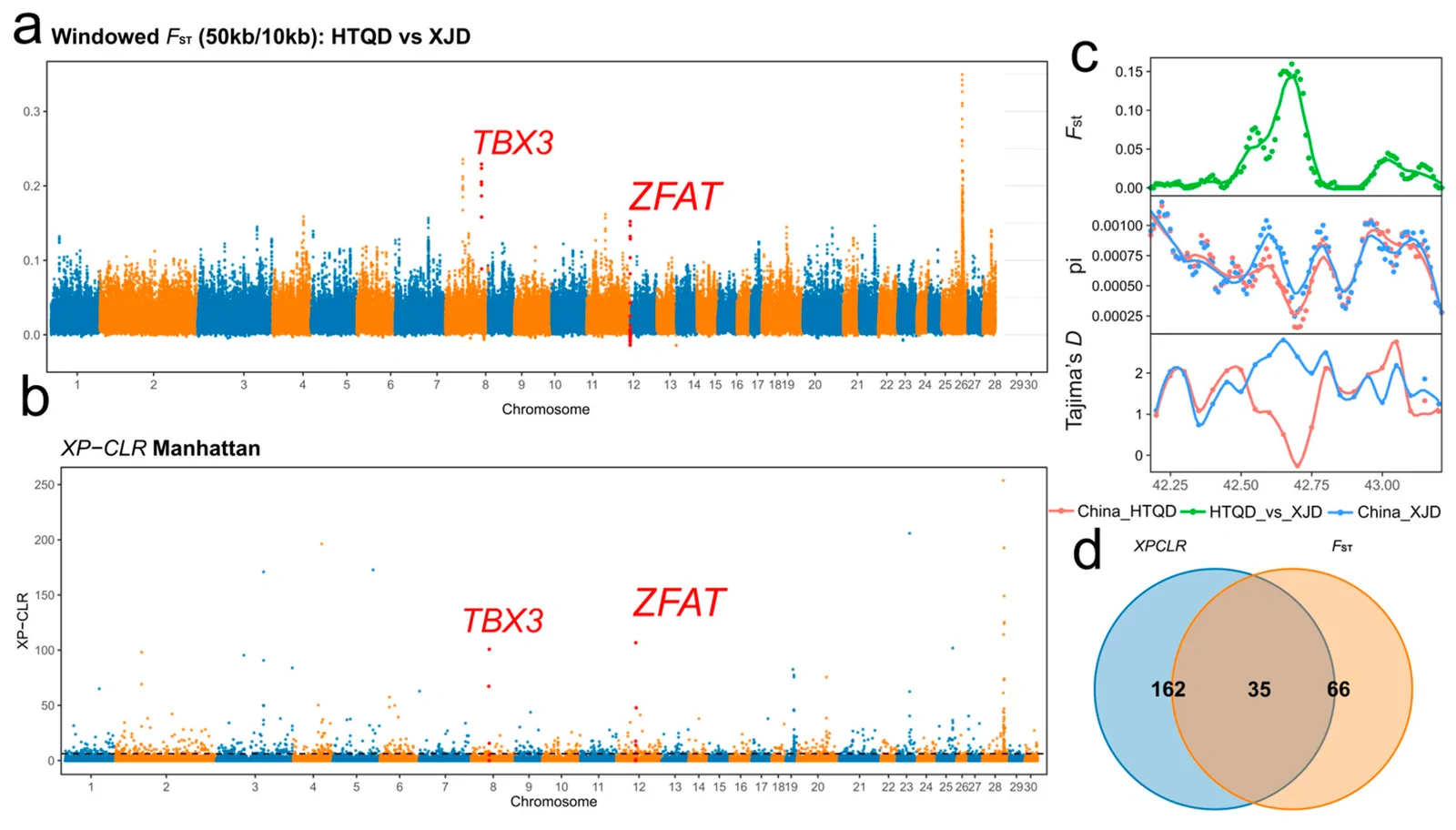
Genome-wide Selection Signal Analysis and Candidate Region Identification between China_HTQD and China_XJD Populations: (**a**) genome-wide scan using Windowed *F*_st_; (**b**) *XP-CLR* scan; (**c**) local statistics profile around *TBX3*; (**d**) overlap of candidate genes for selection signals between the two populations (Venn diagram).

The China_HTQD vs. China_XJD Windowed *F*_st_ analysis (Figure 4a) revealed that the overall genetic differentiation between the two populations was low (below 0.1) at the genomic level, consistent with the previous genetic diversity and structure analyses. However, significant peaks were observed in some chromosomal regions, with distinct clusters near *TBX3* and *ZFAT*, suggesting enhanced population differentiation in these areas, likely related to differential selection or local adaptation. As shown in Figure 4b, the *XP-CLR* method also detected a significant peak corresponding to *TBX3* at the same location as the Windowed *F*_st_ analysis, further confirming that this region has elevated population differentiation and is a strong candidate for selection. Further analysis of the population differentiation and genetic diversity trends within the *TBX3* candidate region for China_HTQD vs. China_XJD (Figure 4c) showed a significant peak in local *F*_st_. Meanwhile, the nucleotide diversity (*π*) near the candidate region in China_HTQD was significantly reduced, accompanied by a negative shift in Tajima’s *D*, consistent with the signature of a selective sweep caused by recent positive selection. Considering both inter-population differentiation and intra-population diversity evidence, this region is a high-confidence candidate for selection signals. The intersection of candidate genes identified by windowed *F*_st_ and *XP-CLR* analyses yielded 35 overlapping genes, of which 2 (*TBX3* and *ZFAT*) were annotated as body size- and coat color-related genes. The remaining 33 genes are listed in Table S5 in the Supplementary Materials. Notably, these two breeds also exhibited significant phenotypic divergence in body-size traits (Table 2), and the identified candidate genes (*TBX3* and *ZFAT*) have been previously implicated in skeletal development and adipogenesis, suggesting that these genetic differences likely underlie the observed phenotypic differentiation.

SNP-level allele frequencies and *F*_st_ values for variants within the *TBX3* locus are presented in Table 5, which lists the top 18 SNPs showing the most pronounced allele frequency differences within this window. For each site, we report its coordinates, reference/alternative alleles, mutation type (transition/transversion/indel), alternative allele frequencies in two populations (HTQD and XJD), and FST. Notably, the highest *XP-CLR* value (56.19) spans all listed variants, while FST reaches a peak of 0.1597 around positions 42,681,707–42,691,755 (overlapping the maximum *XP-CLR* window). Both *XP-CLR* and sliding-window *F*_st_ consistently identified the *ZFAT* region as a strong selection target (peak *XP-CLR* = 8.56; maximum window *F*_st_ = 0.1108). Nevertheless, examination of individual SNPs within this window revealed only modest differences in allele frequencies and no outlier *F*_st_ values (>0.4). Therefore, the selection signal at *ZFAT* is not driven by a single highly differentiated SNP but by a collective shift across many variants.

Thus, while both *TBX3* and *ZFAT* exhibit strong *XP-CLR* signals, their underlying sequence architectures differ: *TBX3* shows a classic ‘outlier SNP’ pattern, whereas *ZFAT* exhibits a more polygenic or soft sweep signature.

**Figure 5:**
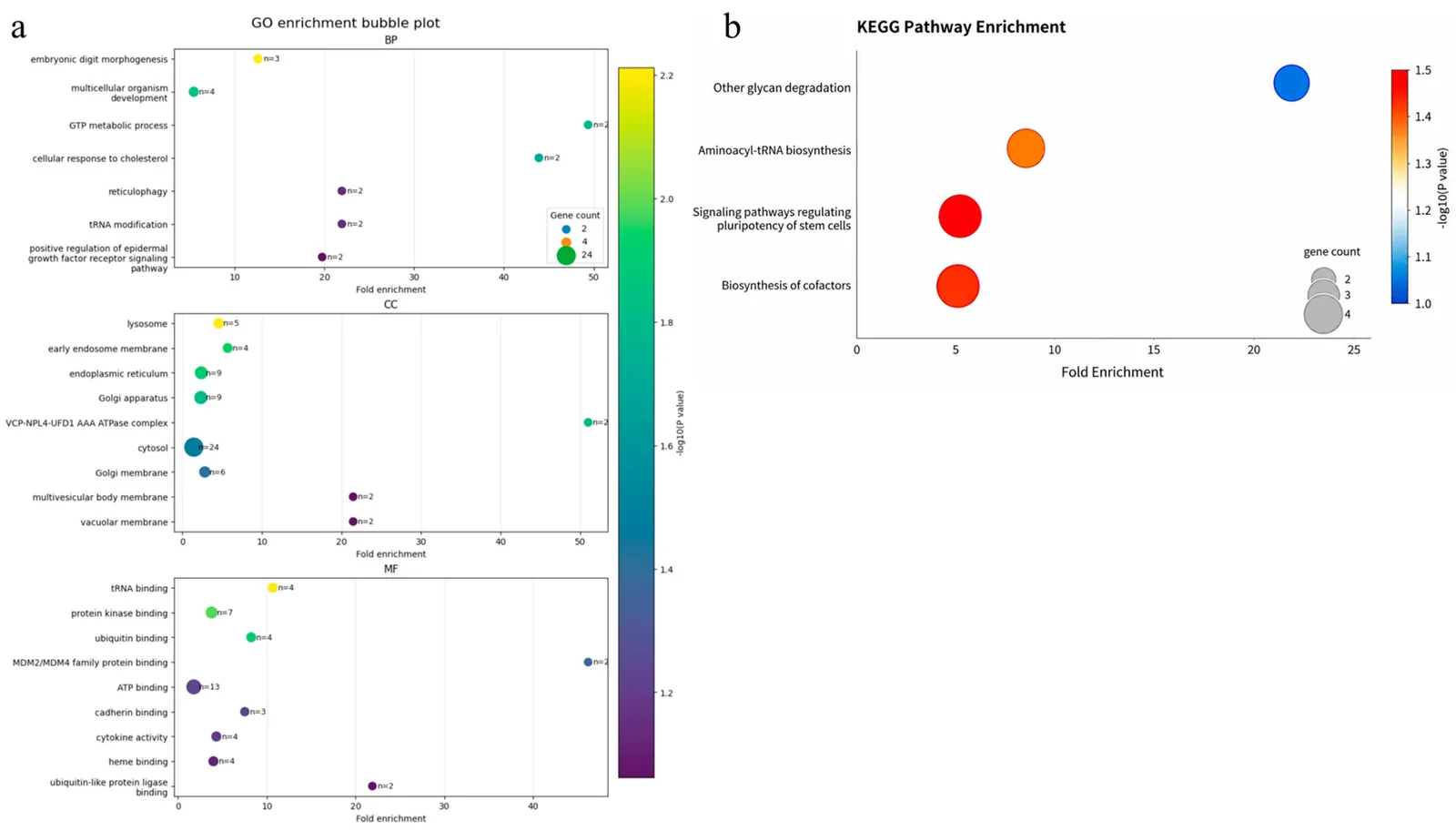
GO and KEGG pathway enrichment bubble plots of candidate genes: (**a**) GO; (**b**) KEGG.

GO enrichment analysis (Figure 5a) further demonstrated that the candidate genes were enriched in seven, nine, and nine GO terms within the biological process (BP), cellular component (CC), and molecular function (MF) categories, respectively. KEGG analysis (Figure 5b) revealed that the candidate genes were significantly enriched in four KEGG pathways (*p* < 0.05), among which *WNT6*, *WNT10A*, *JAK2*, and *TBX3* were mapped to the “Signaling pathways regulating pluripotency of stem cells” pathway. The complete GO and KEGG enrichment results, including all terms, gene counts, and input gene lists, are provided in Table S6. Notably, *TBX3* has been extensively documented in multiple studies as closely associated with coat color and body size traits in donkeys. These candidate genes span diverse functional layers, including developmental regulation, energy metabolism, protein synthesis, and glycan degradation, suggesting that the phenotypic divergence between Xinjiang donkeys and Hetian Gray donkeys likely arises from the coordinated interplay of multiple functional pathways.

## 4. Discussion

### 4.1. Discussion on the Close Relationship and Local Differentiation Features of China_HTQD and China_XJD

In this study, both China_HTQD and China_XJD are located in Xinjiang, China. Given their similar breeding backgrounds, comparisons were made to analyze the genetic distance and degree of differentiation between the two populations by including domestic donkey populations from other regions (other parts of China, Africa, Europe, and West Asia). The PCA, NJ phylogenetic tree, and ADMIXTURE results for the six populations all showed that China_HTQD and China_XJD had very similar genetic distances, indicating similar ancestral compositions and evolutionary histories. In contrast, the European populations exhibited marked genetic distinctiveness, which may be attributed to historical breeding practices, geographic isolation, founder effects, and potential population bottlenecks. This finding also corroborated the result that the genome-wide average *F*_st_ between China_HTQD and China_XJD was low. Meanwhile, the separation of Chinese populations from overseas reference populations in Africa, Europe, and West Asia is consistent with their geographic origins and reflects the broad-scale genetic structure of modern donkey populations, though the observed clustering patterns may be influenced, at least in part, by uneven sampling density between regions—Chinese populations are more densely represented, whereas African and other overseas regions have more limited sample coverage. This not only provided an external reference for positioning China_HTQD and China_XJD but also indirectly supported the reliability of the data quality control and clustering strategy. Although the overall *F*_st_ differentiation level between the two populations was weak, significant peaks of differentiation were observed between chromosomes 8 and 12. This phenomenon has been further confirmed in previous studies. For example, da Silva Ribeiro et al. [30] found that local adaptation or positive selection can occur between populations with “low genome-wide differentiation,” and in such closely related comparisons, differentiation signals often manifest as local peaks. This view further substantiates the conclusion of this study that China_HTQD and China_XJD have close genetic distances, low genome-wide differentiation levels, but significant local differentiation peaks. Additionally, similar viewpoints have been proposed by Booker, T. R. et al. [31]. The regions with significantly elevated *F*_st_ differentiation levels identified in this study can serve as important clues for differential selection between China_HTQD and China_XJD. Kijas et al. [32] found that in multi-breed comparisons, certain breeds (such as Texel) exhibited strong, local differentiation peaks in specific chromosomal regions that matched with breed-defining traits (muscle / growth-related gene regions), exemplifying the pattern of “low differentiation levels but strong local selection peaks,” which further supports the conclusions of this study. The sequencing depth of approximately 10× employed in this study is consistent with the recommended standard for population-level genomic analyses in livestock and has been successfully applied in recent donkey genome-wide studies. Recent methodological assessments indicate that with appropriate parameter settings, sequencing depths as low as 3–5× are sufficient for accurate ROH length and genomic proportion estimation [33]. Accordingly, for ROH detection, we applied optimized PLINK parameters for low-coverage data to minimize potential biases, and *XP-CLR* is inherently less sensitive to moderate depth because it relies on allele-frequency differentiation between populations. Additionally, the intersection of signals from *F*_st_ and *XP-CLR* further enhances the reliability of our candidate gene identification. Nevertheless, several West Central Asian reference populations had limited sample sizes (KAZ = 2, MON = 2, IRA-D = 4, YEM = 3, TUK = 3), and the European samples had 6× coverage. Merging data from different platforms may also introduce batch effects despite stringent QC and LD pruning. In addition, the samples for each of the two Xinjiang breeds were collected from a single breeding farm, which may not fully capture the full genetic diversity across their entire distribution range in Xinjiang. However, since these reference populations were used only for contextualizing population structure and not for selection-signal analyses, and the primary comparisons were performed on consistently sampled populations under controlled conditions, our conclusions remain robust. Future studies with larger samples, deeper coverage, and broader geographic sampling are warranted to validate our findings. Additionally, we envision that future studies with expanded European sampling will enable finer-scale subdivision of this region to further refine population structure analyses.

### 4.2. Reliability Analysis of Phenotypic Differences and Related Candidate Genes Between China_XJD and China_HTQD

Phenotypic measurements of body size and coat color revealed marked differences between the two donkey breeds. This finding is supported by multiple studies; for instance, Haixia, X. et al. [34] reported that Hetian Gray donkeys from Pishan County, Xinjiang, are large-sized, with a withers height of 125–130 cm. In 2025, Li et al. [21] further clarified that Hetian Gray Donkeys, which are representatives of the large-sized breeds in the Hetian region, are closely related to Xinjiang Donkeys but are considered distinct breeds due to “significant phenotypic differences.” Hetian Gray Donkeys are larger in body size, while Xinjiang Donkeys are usually smaller, and genes related to body size have been subject to selection. In this study, the intersection of windowed *F*_st_ and *XP-CLR* selection signals yielded a small set of overlapping genes, including two candidates: *TBX3* and *ZFAT*. Notably, *TBX3* is a critical developmental regulator directly implicated in limb and tissue development [35]. Studies using mouse models have shown that the absence of *TBX3* leads to limb abnormalities and other developmental defects (accompanied by embryonic lethal phenotypes), demonstrating its indispensability in embryonic development [36]. Moreover, *TBX3* participates in establishing key expression boundaries in mouse limb buds through transcriptional repression, thereby affecting limb development programs [37]. These related studies demonstrate that the *TBX3* gene may be involved in regulating body size traits in China_XJD and China_HTQD and is a key candidate gene underlying differences in body size between the two populations. *TBX3* showed a robust selection signal (*XP-CLR* peak = 56.19) and a well-established role in limb and tissue development [38,39,40]. While Li et al. [21] identified *TBX3* as a breed-differentiating candidate, our study further pinpoints *ZFAT* as a novel candidate and provides the first testable hypotheses for its functional role in coat color variation. In contrast, *ZFAT* exhibited a considerably weaker signal (*XP-CLR* peak = 8.56), with no outlier SNPs detected within this window, suggesting a more tentative role. Although *ZFAT* has been implicated in body size traits in other livestock [41], its potential involvement in coat color regulation remains highly speculative, based primarily on its hematopoietic function and indirect modulation of Gata1 [42,43] rather than direct evidence in equids. We therefore propose *ZFAT* as a secondary candidate, with its possible contributions to phenotypic divergence, particularly coat color, requiring further investigation through targeted expression and functional studies. Future studies incorporating GWAS, allele effect estimation, haplotype analysis, and functional experiments are needed to validate the causal roles of these candidate genes.

## 5. Conclusions

This study demonstrates that despite their highly similar genetic backgrounds, Hetian Gray Donkeys and Xinjiang Donkeys—two closely related local breeds—maintain substantial genetic diversity. Through *F*_st_ and *XP-CLR* selection signal analyses, we identified significant genetic differentiation in specific genomic regions and pinpointed *TBX3* and *ZFAT* as candidate genes under selection. Enrichment analysis further confirmed their critical roles in phenotypic divergence between the two populations. *TBX3* emerged as the primary candidate with a strong selection signal and a well-established role in body size regulation, whereas *ZFAT*, with a weaker signal, is proposed as a secondary candidate, with its potential involvement in coat color representing a speculative hypothesis requiring direct experimental validation in future studies. These findings provide molecular insights into the genetic mechanisms underlying their phenotypic differences and establish a scientific foundation for conserving and utilizing Xinjiang’s local donkey genetic resources.

## Figures and Tables

**Table 1:** Sample information for the modern donkey whole-genome dataset.

Region	Variety Name	Abbreviation of Variety Name	DataSource	*N*
China	Hetian Gray donkey	China_HTQD	SELF	30
	Xinjiang donkey	China_XJD	SELF	30
	Sichuan donkey	CL	NCBI	11
	Guangling donkey	GLD	NCBI	18
	Huaibei Gray donkey	HB	NCBI	13
	Tibet donkey	XZCM	NCBI	18
	Yangyuan donkey	Yangyuan	NCBI	22
Africa	Egyptian donkey	EGY	NCBI	9
	Ethiopian donkey	ETH	NCBI	9
	Kenyan donkey (YPO population/site)	KEN-YPO	NCBI	18
	Somali donkey	SOM	NCBI	4
Europe	Canary Islands donkey	CYK-IslasCanarias	NCBI	5
	English White & Pure Irish donkey	EnglishWpureIrish	NCBI	5
West Central Asia	Iranian donkey	IRA-D	NCBI	4
	Kazakhstan donkey	KAZ	NCBI	2
	Kyrgyzstan donkey	KYR	NCBI	9
	Mongolian donkey	MON	NCBI	2
	Yemen donkey	YEM	NCBI	3
	Turkmenistan donkey	TUK	NCBI	3

Note: “SELF”: self-test data. “China_Ref” comprises five Chinese donkey populations (Sichuan, Guangling, Huaibei Gray, Tibet, Yangyuan) and serves solely for visualization and reference purposes.

**Table 2:** Major phenotypic differences between Xinjiang donkey and Hetian Gray donkey.

Breed	Trait	Mean ± SD	Max	Min	CV (%)
China_XJD	BW (kg)	169.27 ± 15.70 ^a^	201	145	9.28
	BH (cm)	101.53 ± 3.89 ^a^	109	95	3.83
	BL (cm)	104.97 ± 3.93 ^a^	112	97	3.75
	CC (cm)	108.67 ± 4.53 ^a^	118	100	4.17
	CaC (cm)	13.20 ± 0.86 ^a^	15	11.8	6.51
	Coat color	Gray, Black			
China_HTQD	BW (kg)	238.93 ± 20.67 ^b^	277	205	8.65
	BH (cm)	129.63 ± 4.30 ^b^	137	122	3.32
	BL (cm)	132.60 ± 5.16 ^b^	143	124	3.89
	CC (cm)	139.17 ± 5.45 ^b^	149	131	3.91
	CaC (cm)	13.63 ± 0.8 ^b^	15.5	12.5	6.01
	Coat color	Cyan (blue-gray)			

Note: “BW”: Body weight, “BH”: Body height, “BL”: Body length, “CC”: Chest circumference, and “CaC”: Cannon circumference. Within the same trait, different superscript letters (a, b) indicate significant differences between breeds (*p* < 0.05); identical letters or absence of letters indicate non-significant differences. Letters are ranked by mean value (a > b).

**Table 3:** Quality Control of Sequencing Data and Alignment Results with the Reference Genome.

Group	Cleanreads	Sequencing Depth (×)	Mapping Rate /%	SNP Density /kb	Coverage Ratio (10×)	GC (%)
China_HTQD	170631025	8	97	4.452502	89.57	41.81
China_XJD	180114408	9.6	98	4.392043	95.73	41.68
China_Ref	110143213	9.1	97	4.930633	93.89	42.14
Africa	121539402	8.7	95	4.606308	91.46	42.69
West Central Asia	172038524	10.8	97	4.122781	97.86	42.91
Europe	98392037	6	96	2.744049	68.15	41.02

Note: “China_Ref” represents the collective designation for samples from other regions of China (Sichuan donkey, Guangling donkey, Huaibei gray donkey, Tibet donkey, Yangyuan donkey). This combined group is used only for visualization purposes and does not represent a single breed.

**Table 4:** Genetic Diversity Indices of Hetian Gray Donkeys (China_HTQD), Xinjiang Donkeys (China_XJD), and Other Reference Populations.

Group	Nucleotide Diversity (*π*)	Median_NROH	Median_TotalROH (Kb)	Median Tajima’s *D*	*PIC*	*Ho*	*He*	*F_ROH_* (%)
China_HTQD	0.000612	50	36467	1.26	0.252373	0.103799	0.127063	0.0152
China_XJD	0.000613	54.5	41224	1.27	0.252344	0.109425	0.128111	0.0172
China_Ref	0.000516	84	74335	1.68	0.246124	0.109794	0.128089	0.0310
Africa	0.000602	104	83341	1.48	0.250682	0.10237	0.127465	0.0347
West Central Asia	0.000608	63	50567	1.10	0.255015	0.077307	0.125038	0.0211
Europe	0.000572	220	264551	0.803	0.27189	0.113682	0.127711	0.1102

**Table 5:** SNP-level allele frequencies and *F*_st_ values in the *TBX3* candidate region.

Gene	CHROM	POS	Ref	Alt	Type	China_HTQD altFreq	China_XJD altFreq	Single_Snp_*F*_st_ Value
*TBX3*	8	42671290	C	G	Transversion	0.8000	0.3667	0.3133
		42673941	C	T	Transition	0.8167	0.2500	0.4306
		42675259	A	G	Transition	0.8000	0.4000	0.3112
		42677528	A	G	Transition	0.1500	0.5500	0.3017
		42681707	C	G	Transversion	0.1833	0.5833	0.3288
		42682049	A	G	Transition	0.2000	0.6167	0.2942
		42682205	G	A	Transition	0.1667	0.5500	0.3003
		42684924	C	T	Transition	0.1500	0.5667	0.3040
		42687155	C	A	Transversion	0.1833	0.5833	0.3025
		42688252	C	G	Transversion	0.0667	0.4667	0.3696
		42688954	T	C	Transition	0.0500	0.5000	0.3831
		42689240	T	C	Transition	0.1833	0.6000	0.2971
		42689963	G	A	Transition	0.0833	0.5167	0.3558
		42698414	A	G	Transition	0.1833	0.6000	0.2981
		42700904	G	A	Transition	0.1833	0.6000	0.2971
		42702524	C	G	Transversion	0.0667	0.5000	0.3860
		42717346	T	A	Transversion	0.7833	0.2833	0.4277
		42719160	C	T	Transition	0.6333	0.1833	0.4444

Note: “CHROM” indicates the chromosome, “POS” denotes the position of the SNP, “Ref” is the reference allele, “Alt” is the alternative allele, and “altFreq” represents the frequency of the alternative allele.

## Data Availability

The raw sequencing data generated in this study have been deposited in the Genome Sequence Archive (GSA) at the National Genomics Data Center, China National Center for Bioinformation, under accession number CRA046128 (Project ID: PRJCA068742).

## Supplementary Materials

The following supporting information can be downloaded at: https://www.mdpi.com/article/10.3390/ani16182825/s1, Figure S1: Locus missing rate; Figure S2. Sample missing rate, MAF, and heterozygosity; Figure S3. LD decay. Table S1. Metadata of 155 publicly available donkey genomes downloaded from NCBI. Table S2. Quality control statistics. Table S3. Summary of per-population sample missingness rates (F_MISS) after quality control filtering. Table S4. Pairwise genome-wide *F*_st_ values. Table S5. Summary of all candidate genes detected by both *F*_st_ and *XP-CLR* analyses. Table S6. GO_KEGG_enrichment_terms_adjustedP_genecount_background_input(1).

## Abbreviations

The following abbreviations are used in this manuscript:

China_HTQD	Hetian Gray Donkey population in China
China_XJD	Xinjiang Donkey population in China
China_Ref	Other Chinese reference donkey populations
PCA	Principal Component Analysis
NJ	Neighbor-Joining
LD	Linkage Disequilibrium
ROH	Runs of Homozygosity
*F_ROH_*	Inbreeding coefficient based on ROH
*Ho*	Observed Heterozygosity
*He*	Expected Heterozygosity
*PIC*	Polymorphism Information Content
*π*	Nucleotide Diversity
*F*_st_	Fixation Index (Genetic Differentiation Statistic)
*XP-CLR*	Cross-Population Composite Likelihood Ratio
SNP	Single Nucleotide Polymorphism
NCBI	National Center for Biotechnology Information
*TBX3*	T-Box Transcription Factor 3 gene
*ZFAT*	Zinc Finger and AT-Hook Domain Containing gene
